# Why Is Whipple's Disease Still a Challenging Diagnosis? A Case Report and Brief Review of Literature

**DOI:** 10.7759/cureus.34029

**Published:** 2023-01-21

**Authors:** Ana Filipa Viegas, Andreia M Lopes, Gabriela Venade, Pedro Rodrigues, João Tavares

**Affiliations:** 1 Department of Internal Medicine, Centro Hospitalar Tondela-Viseu, Viseu, PRT; 2 Department of Anatomic Pathology, Centro Hospitalar Tondela-Viseu, Viseu, PRT

**Keywords:** polymerase chain reactions, periodic acid-schiff staining, undiagnosed diseases, delayed diagnosis, whipple disease

## Abstract

Whipple's disease (WD) is a rare multisystemic infectious disease caused by *Tropheryma whipplei.* The pathogenesis of Whipple's disease remains unknown and clinical experience relies solely on various case reports published in the literature. The disease may occur at any age, with most studies describing patients in their fifth decade. Classic WD mainly affects the gastrointestinal tract, but extraintestinal commitment can occur, with the most common manifestations being arthralgias, lymphadenopathy, fever, and neurological symptoms.

We present a case of a 69-year-old woman who presented with fever, macular rash, abdominal pain, lymphadenopathy, pleural and pericardial effusion, weight loss, and severely altered mental status over seven days. Initial workup tests only revealed leucopenia, thrombocytopenia, and hyperferritinemia. Since the fever persisted despite antibiotic treatment, an extensive workup was required until the final diagnosis of classic WD through histological examination of duodenal biopsies. Treatment with ceftriaxone was implemented for two weeks, followed by trimethoprim-sulfamethoxazole 160/800mg bid for 12 months. The patient presented full recovery and no recurrence after three years of follow-up.

Even though WD was first described more than a century ago, WD is an elusive disease with a wide variety of clinical findings, leading to a still significant delay in diagnosis. WD should be considered in the differential diagnosis of rheumatologic disorders, chronic abdominal pain or diarrhea, neurological manifestations not suggestive of any other specific disease, non-caseating granulomatous diseases, and cases of lymphadenopathies. The authors aim to add additional clinical data and raise awareness for a rare condition that can be lethal if not timely treated. More studies and recommendations are needed concerning screening patients and treatment, with an urgent need to improve the delay in diagnosis.

## Introduction

Whipple's disease (WD) is a chronic multisystemic infectious disease caused by the rod-shaped, gram-positive bacteria *Tropheryma whipplei *and with an annual incidence of 1-6:1000000 [1]. It has a male predominance with a ratio of 8:1, and the diagnosis is usually made between the fifth and sixth decade of life, even though it can occur at any age [1-3]. The pathogenesis remains unclear, but host immunity appears to play a role in developing the disease, with macrophage function and type-1 T-cell response impaired in symptomatic individuals [4].

The clinical presentation is very heterogeneous, but there are four recognized patterns of infection: Classic WD, localized chronic infections, acute infections, and asymptomatic carriers [5]. Classic WD mainly affects the gastrointestinal tract, especially the small intestine, presenting with abdominal pain, diarrhea, steatorrhea, and weight loss. Still, extraintestinal commitment can occur with the potential involvement of every organ. The most common extraintestinal manifestations are arthralgias, lymphadenopathy, fever, and neurological symptoms [1].

The disease was first described in 1907 by George Hoyt Whipple. There is usually a substantial delay between the onset of symptoms and the diagnosis of the disease, and many cases are misdiagnosed as rheumatic diseases [2,5]. When WD is suspected, diagnosis can be confirmed through biopsy specimens collected from duodenal or other involved tissues, in which positivity for periodic acid Shiff (PAS) staining and non-acid-fast in Ziehl-Neelsen staining are seen [2]. In the last decades, diagnosis has become more accessible due to the discovery of polymerase chain reaction (PCR), which can be used in various biological samples [1].

Herein we report a case of classic WD that had a challenging diagnosis, with a complex variety of symptoms related to gastrointestinal, neuropsychiatric, hematologic, and serosa involvement.

## Case presentation

A 69-year-old female presented to the Emergency Department with a one-week history of persistent fever (three spikes per day with a maximum temperature of 39ºC), generalized macular rash, and weight loss (not quantified). In addition, she mentioned chronic constipation and abdominal pain in the upper quadrants, also exacerbated in the prior week. The patient denied arthralgia or other symptoms, but her relatives stated that progressive episodic behavioral changes, loss of memory, and increasingly more difficulty with everyday tasks had been noted for the previous four months. Her medical history included dyslipidemia, depression, and hypothyroidism, under treatment with simvastatin 20mg od, paroxetine 20mg od, and levothyroxine 0.125mg od. She lived in the countryside and had no animals or other relevant epidemiological history.

At physical examination, she was febrile (38.5ºC), hemodynamically stable, with painful abdominal palpation on the epigastric area and left flank, without signs of peritoneal irritation. An evanescent macular rash was present in the trunk, abdomen, and thighs, particularly noticed in fever spikes. Neurological examination was normal, and the remainder of her examination was unremarkable. Her first laboratory results revealed mild leucopenia with lymphopenia, slight thrombocytopenia, and increased creatinine kinase, lactate dehydrogenase, aspartate/alanine aminotransferases, and C-reactive protein (Table 1). Abdominal ultrasound showed slight dilatation of the common biliary duct, with no visible site of obstruction. A magnetic resonance (MRI) cholangiopancreatography later confirmed a small dilatation of the common biliary duct without lithiasis or suspected masses.

**Table 1:** Initial laboratory results

Initial laboratory results	Results	Normal Range
Leucocytes	3.10 G/L	4.5-11.5 G/L
Hemoglobin	13.2 g/dL	12-15 g/dL
Mean Globular Volume	83 fL	81-97 fL
Platelets	112 G/L	150-450 G/L
C-reactive protein	9.07 mg/dL	<0,5 mg/dL
Procalcitonin	0.14 ng/mL	0.01-0.64 ng/mL
Blood Urea	40 mg/dL	16-42 mg/dL
Serum Creatinine	0.9 mg/dL	0.5-1.2 mg/dL
Serum Ionogram	Normal	-
Serum total protein	5.8 g/dL	6.6-8.7 g/dL
Serum albumin	3.6 g/dL	3.5-5 g/dL
Creatinine kinase	365 UI/L	34-145 UI/L
Lactate dehydrogenase	1517 UI/L	200-480 UI/L
Aspartate/alanine aminotransferase	92/75 UI/L	13-31 UI/L
Gamma-glutamyl transferase, alkaline phosphatase	30/68 UI/L	4-32/25-100 UI/L
Serum amylase/lipase	48/50 UI/L	8-53/6-51 UI/L
Serum total bilirubin	0.7 mg/dL	0.2-01 mg/dL
Urinalysis	Normal	-
Urine culture	Negative	-
Blood cultures	Negative	-

She was admitted to the Internal Medicine Department with a possible diagnosis of zoonosis and started treatment with doxycycline 100mg bid and ceftriaxone 2g od. On the first days after admission, she presented signs of severe cognitive impairment and erratic behavior that fluctuated throughout the day. A brain computed tomography (CT) was obtained and was normal, followed by a lumbar punction which revealed a clear cerebrospinal fluid (CSF), with slight proteinorrhaquia and without pleocytosis, as well as a negative microbiological panel by PCR detection (Table 2). After one week of admission, the rash and abdominal pain improved, but fever and delirium persisted. The results of blood, urine, and CSF cultures previously obtained were all negative (Tables 1, 2). Additionally, an electroencephalogram revealed slight cerebral dysfunction and encephalopathy grade 1, and brain magnetic resonance imaging (MRI) had no alterations.

**Table 2:** Cerebrospinal fluid analysis results CSF: cerebrospinal fluid; PCR: polymerase chain reaction

Cerebrospinal fluid analysis results	Results	Normal Range
Cells	4 leucocytes/mm^3^	-
Proteins	46.9 mg/dL	15-40 mg/dL
Glucose	55 mg/dL	50-80 mg/dL
Multiplex panel by PCR detection - *Escherichia coli, Haemophilus influenzae, Listeria monocytogenes, Neisseria. meningitidis, Streptococcus agalactiae, Streptococcus pneumoniae, Cytomegalovirus, Enterovirus, Herpes Simplex virus 1, 2 and 6, Human Oarechovirus, Varicella Zoster Virus and Cryptococcus neofarmans/gatti*	All negative	-
PCR *Mycobacterium tuberculosis*	Negative	-
Culture of CSF	Negative	-

An extensive and sequential complementary study was performed. She maintained mild-to-moderate leukopenia, mild thrombocytopenia, and elevated C-reactive protein, with a persistently normal erythrocyte sedimentation rate and procalcitonin, but elevated ferritin (1332 ng/dL, normal range 10-291 ng/mL). Multiple serologic studies, an autoimmunity panel, serum immunoglobulins levels, and C3/C4 levels were executed, all in the normal range (Table 3). A transthoracic echocardiogram was also performed with no apparent vegetation or other alterations, and thorax X-ray and urinalysis were continuously normal. After two weeks of admission and faced with persistent clinical features and an extensive but inconclusive etiologic study, a positron emission tomography-CT (PET-CT) scan with 18F-fluorodeoxyglucose (18F-FDG) was made and revealed a low-volume bilateral pleural effusion, discrete pericardial effusion, abdominal lymphadenopathies without significant uptake, increased uptake of the shoulders bilaterally and hypermetabolic fixation on the axial skeleton and spleen. There was also an increased uptake on the stomach and small bowel, presumably related to normal physiological activity.

**Table 3:** Other laboratory results ANA: antinuclear antibodies; anti-dsDNA: anti-double stranded deoxyribonucleic acid; anti-MPO: anti-myeloperoxidase; anti-PR3: anti-proteinase 3; C3: complement component 3; C4: complement component 4; ENA: extractable nuclear antigens

Other laboratory results	Results	Normal Range
Serum Ferritin	1332 ng/mL	10-291 ng/mL
Transferrin saturation	10.25%	20-50%
Serum B12 vitamin levels	547 pg/mL	179-1130 pg/mL
Serum Folic acid levels	7.2 ng/mL	1-20 ng/mL
Erythrocyte sedimentation rate	6 mm/s	0-20 mm/s
Total cholesterol	211 mg/dL	<200 mg/dL
Triglycerides	267.4 mg/dL	<150 mg/dL
Serologic studies *- Brucella, Salmonella, B and C hepatitis, Cytomegalovirus, Epstein-Barr Virus, Leptospira, Treponema pallidum, Mycoplasma pneumoniae, Human immunodeficient virus 1 and 2, Rickettsia conorii, Coxiella burneti, Wright reaction and Widal reaction*	All negative	-
Autoimmunity panel - ANA, ENA screen, anti-dsDNA, Anti-MPO, Anti-PR3	All negative	-
Serum Immunoglobulin levels	Normal	-
C3/C4 complement levels	122.4/19.3 mg/dL	83-177/12-36 mg/dL

**Figure 1:**
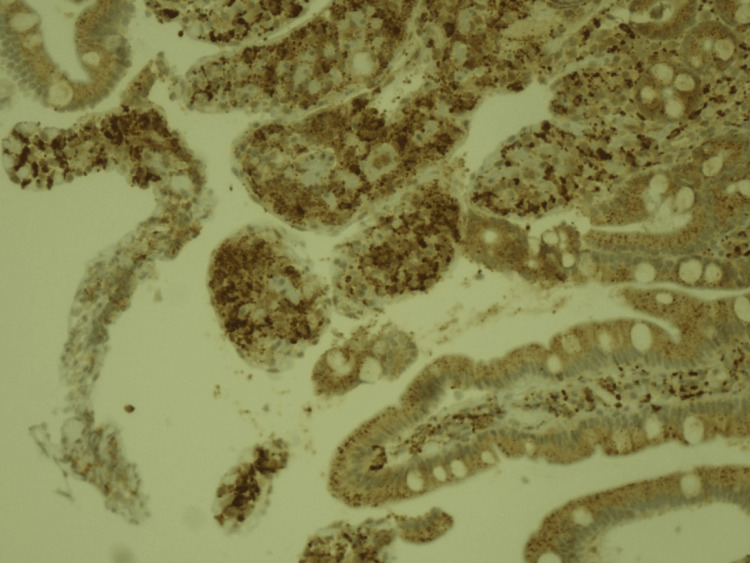
Immunohistochemical staining of duodenal biopsy specimens with inflammatory infiltrate with CD68 positive histiocytes (200X lens objective)

**Figure 2:**
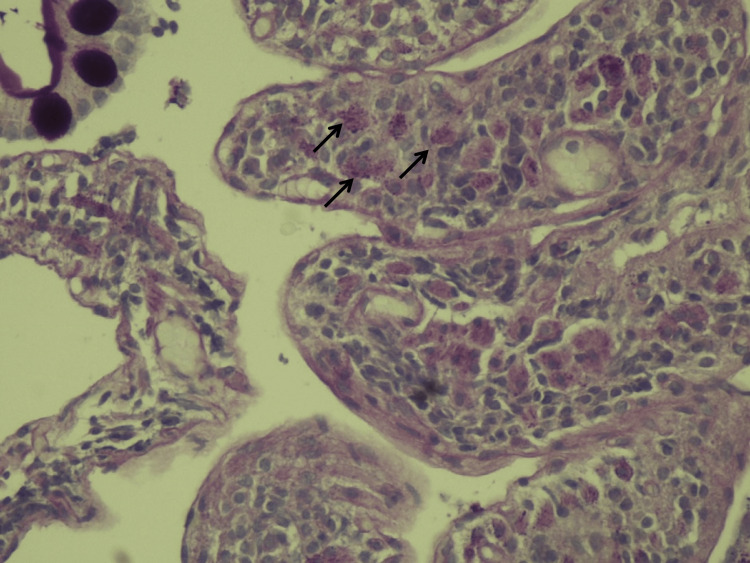
PAS-stained duodenal biopsy specimens with eosinophilic granules inside macrophages PAS-positive - arrows (400X lens objective) PAS: periodic-acid schiff

A multidisciplinary meeting occurred, and the possibility of Still's Disease with possible macrophagic activation syndrome and WD were postulated as likely hypotheses. A bone biopsy and myelogram revealed hypercellular bone marrow without hemophagocytosis. Upper gastrointestinal endoscopy showed subtle duodenal lymphangiectasis, and duodenal biopsies were made to exclude WD. Biopsy results confirmed WD on the 37^th^ day of admission, showing enlargement of duodenal villi with inflammatory infiltrate with histiocytes and with eosinophilic granules inside macrophages PAS-positive (Figures 1, 2). PCR for *T. whipplei* was not possible due to technical limitations.

She restarted treatment with ceftriaxone 2g od for two weeks and switched to sulfamethoxazole-trimethoprim 960mg bid at the time of discharge, with clinical and analytical improvement. The patient maintained sulfamethoxazole-trimethoprim for 12 months, with a full cognitive recovery and normalization of hemogram, ferritin, and C-reactive protein values. An upper gastrointestinal endoscopy was repeated after one year and was utterly normal in macroscopic and microscopic evaluation, with negative PCR for *T. whipplei* in duodenal biopsies. After three years of follow-up, no signs of recurrence were seen.

## Discussion

This report presents a classic WD case with gastrointestinal, neuropsychiatric, hematologic, and serosa involvement. The diagnosis was laborious and challenging, and a high suspicion of Whipple Disease was essential. WD is a rare and heterogeneous disease related to multisystemic involvement and non-specific symptoms. The diagnosis can be difficult, and the average time between the first symptoms and diagnosis is broad, ranging from 3-7 years, due to low grade of suspicion in daily clinical practice [2,3,6]. The typical course of classic WD begins with an early phase in the first six years, with intermittent arthralgias and fever, followed by an intermediate phase with gastrointestinal symptoms. After over eight years, the late phase occurs with predominant neurological symptoms [7].

Gastrointestinal symptoms are the most classical feature of WD and are the most common at the time of diagnosis [2]. WD usually affects the lamina propria of the small intestine and its lymphatic drainage, with 72%-81% of patients having diarrhea and 23%-60% with abdominal pain, which is usually epigastric or diffuse [1-3,6,8]. Less frequently, patients have constipation (1.4%-7%), ascites (4%-7%), and gastrointestinal bleeding (2%-3%) [3,6,8]. Constitutional symptoms are also common, including weight loss (44.2%-99%), fever (19%-54%), anorexia (15.6%), and asthenia (6.1%) [1-3,6,8].

Arthralgias are seen in 20%-83% of patients, commonly as the first symptom, and usually precede the diagnosis several years [1-3,6]. The patients frequently have an oligoarticular or polyarticular involvement with a symmetric, intermittent and migratory pattern [5-7]. The most affected joints are the wrists, knees, ankles, hips, shoulders, and interphalangeal joints [9]. Axial involvement can also be seen in rare cases [3,7]. It is important to emphasize that around 50% of patients with WD are first incorrectly diagnosed with an inflammatory rheumatologic disease and start immunosuppressive treatment. In these cases, the absence of clinical improvement or exacerbation of symptoms after initiating immunosuppressive treatment is common and should raise the suspicion of WD or other alternative diagnoses [2,3,5].

The prevalence of neurological involvement is 6%-63% [1-3,6], presenting with confusion or coma due to meningoencephalitis or status epilepticus, cognitive impairment with memory loss and attention deficit, pyramidal, and extrapyramidal symptoms, sleep disorders, cerebellar palsy, abnormal involuntary movements (including oculomasticatory or oculofacial-skeletal myorhythmia), oculomotor nerve palsy and progressive dementia [7]. Analysis of CSF reveals normal cytology in around half of the patients with neurological symptoms, with the remainder of cases having a CSF compatible with lymphocytic meningitis, with normal or high protein levels [7,8,10]. Despite this, detection of *T. whipplei* through PCR seems to be as high as 41%-63% of all cases of classic WD who underwent lumbar punction, even without neurological symptoms [2,10,11]. If neurologic symptoms are present, up to 59.1%-92% have a positive PCR for *T. whipplei *[2,7,10]. Brain imaging can be normal or show focal or multifocal lesions, particularly in a supratentorial localization. Additionally, periventricular diffuse leukopathy, diffuse cortical atrophy, and pachymeningitis can be seen. Brain MRI seems to have a better performance than CT in detecting alterations [7,8,10]. In this case report, neurological involvement was pronounced during admission, and no other etiology was found. MRI was normal, and unfortunately, PCR on CSF wasn't possible to perform at the time, but the full recovery after treatment supports the assumption of neurological involvement from WD.

Adenopathies are seen in 18.4%-66% of patients, and it is mandatory to exclude lymphoma in these cases [1,3,6,8]. Peripheral and mesenteric localizations are the more frequent [3,6], which is compatible with the localization seen in our patient. In several cases, the diagnosis of WD is made through histological examination of a lymph node that shows granulomas with PAS-positive inclusions [12]. Peripheral blood changes are frequently seen, especially anemia (75%-90% cases), leukocytosis, and thrombocytosis [1-3]. Leucopenia with lymphopenia is rarely described, and thrombocytopenia, when present, is frequently related to hypersplenism [8,13]. Bone marrow involvement is not often reported; however, it is presumably underestimated since bone marrow examination isn't commonly performed. Previously reported cases in which bone marrow was examined and compatible with WD showed focally disrupted microarchitecture with non-caseating granulomas consisting of histiocytes, epithelioid cells, lymphocytes, and macrophages/histiocytes with PAS-positive inclusions. Other findings include increased mast cells, eosinophils, and reticulin fibers around the granulomas [13-15]. In our case, leucopenia and thrombocytopenia were present, which are rare but similar to the case reports from Tun et al. [14] and Rausing et al. [13]. In these two reported cases, a bone marrow examination confirmed the diagnosis of WD, even though in the Rausing et al. case report, it was only possible post-mortem and after reviewing the histology years later [13,14]. Unfortunately, PAS staining was not performed on our patient's bone marrow. Hepatomegaly and splenomegaly are also described in 15% and 6% of patients, respectively [6]. Hepatic and spleen biopsies of WD patients usually reveal non-caseating granulomas and macrophages with PAS-positive inclusion [13,14].

Cutaneous manifestations are seen in around 12.2%-32% [6,8]. The most common alteration described is skin hyperpigmentation associated with severe malabsorption. There also are reported cases of purpura, subcutaneous nodules, and macular rash, as seen in our patient, despite not being frequently seen [6]. Uveitis, commonly related to rheumatologic disorders or bowel inflammatory disease, is also reported in 3.4% [8] and should be remembered as a possible manifestation of WD. Lung and heart involvement with myocarditis, pericarditis, or endocarditis is also reported, with endocarditis and pneumonia being mostly seen in localized WD [1]. The prevalence of pleural effusion is 1.4%-12% [3,6] and pericardial effusion is 1.6%-2.7% [6,8]. Our patient also presented pleural and pericardial effusions, resolved after adequate treatment and without any alternative diagnosis of primary heart or lung disease.

The diagnosis of WD is classically confirmed by typical histological findings and, in recent years, supported by the use of PCR that detects *T. whipplei *in multiple samples [1,2]. Upper gastrointestinal endoscopic features can be normal in up to 74%, and some macroscopic findings can be indicative, but not pathognomonic, of WD, such as duodenal clumsy or dilated villi, ecstatic lymph vessels, edema, and duodenitis [2]. Histological features include typical foamy macrophages with granular diastase-resistant inclusions presenting a strong positive PAS reaction and can reveal villous atrophy, lymphangiectasia, and intramucosal lipid deposits. Multiple biopsies in different sites should be performed to increase sensitivity [2,16]. The use of antibiotics previously to biopsies can cause a weaker PAS reaction, which Herbay et al. [16] classified as subtypes 2 and 3 of PAS-positive macrophages. This is probably the situation of our patient, that had previously been under treatment with ceftriaxone 2g for eight days. Immunohistochemistry (IHC) using antibodies directed explicitly against the bacteria can also increase specificity and sensitivity, especially to differentiate from *Mycobacterium* infections that can also be PAS-positive [5]. Electron microscopy observation shows lysosomes filled with numerous rod-shaped bacteria [16].

The performance of PCR in duodenal biopsies to confirm the presence of *T. whipplei* also increases specificity and has a sensitivity of 96% for classic WD [3] and 72.6% for WD in general [11]. Detection of *T. whipplei* in stools through PCR has a sensitivity of 81%-84% and specificity of 97.6% [3,11]. However, its positive predictive value (PPV) in feces is low (81%) [11], which can be justified by an estimated prevalence of 2%-4% positivity in the stools of asymptomatic carriers. The PPV becomes higher (95.2%) if the patient has a combined positive PCR in stools and saliva or if the bacterial load in feces is higher, with a proposed threshold of >32 200 copies/mL [11]. Concerning saliva alone, its sensitivity is lower than in stools (57.7%-68%), but the PPV is higher (96%) [3,11]. Furthermore, Moter et al. [17] showed that 9 of 12 patients with classic WD had positive PCR in urine samples, whereas controls were all negative, even if PCR was positive in the stools of asymptomatic carriers. In addition, Fenollar et al. [11] reported a PPV and specificity of 100% on urine samples, however, with a sensitivity of 37.5%. In this sequence, some experts recommend the initial use of PCR in stools and saliva to screen suspected WD patients [11], and in the future, possibly the use of urine samples will be recommended too; since it has a higher PPV, but more data are needed. Regarding CSF, PCR has a 100% specificity and is commonly positive (41-63%), even in patients without neurological symptoms. However, sensitivity is low due to the scarcity of *T. whipplei* in CSF, which could make the confirmation of neurological involvement harder [2,3,11]. Moreover, PCR performance on blood appears to have low sensitivity (36.9-52%) [3,11], so its use is not commonly recommended. Unfortunately, PCR was unavailable in our center at the time of the diagnosis, so it was impossible to perform it.

Concerning acute phase reactants, increased C-reactive protein is seen in 69%-84% of patients [1,2], but increased ESR is more uncommon (1.6%) [6]. The prevalence of increased ferritin was not found in the reviewed literature on WD, but the authors postulate a probable relation to WD since its value only normalized after proper treatment of WD. This increase probably reflects ferritin's role as an acute phase reactant since pro-inflammatory cytokines stimulate the synthesis of ferritin and hepcidin, leading to hyperferritinemia, iron retention in macrophages, and less available iron for erythropoiesis due to body redistribution [18]. 

No imaging exam is specific for the diagnosis of WD, and its use varies according to clinical presentation [5]. The role of PET-CT in WD is not established, but recently it has been suggested for the initial evaluation and follow-up of WD chronic infections. Kadian et al. [19] described enlarged low-attenuation lymph nodes in the root of the mesentery and retroperitoneum without significant 18F-FDG uptake, as seen in this case report. In addition, Lagier et al. [20] found increased 18F-FDG uptake in the small bowel in a case of WD endocarditis with gastrointestinal relapse, which was not seen in the first PET-CT performed. More studies are needed to better understand PET-CT scan's role in diagnosing WD and if it can give precocious clues to aid an earlier diagnosis.

Without adequate treatment, WD can have irreversible sequels and be fatal, even though mortality rates are unknown. Antibiotic treatment usually leads to rapid improvement, but an extended duration of antibiotics is required to eradicate the bacteria. Relapses appear in up to 30% of cases; however, they have declined over the last decades. Eradication is more challenging if eye, heart, or neurological involvement is present, with a higher proportion of relapse and mortality in these cases [6,15]. The most usual antibiotic regimen seen is the administration of intravenous ceftriaxone 2g od or meropenem 1g tid (if ceftriaxone isn't tolerated) for the first 14 days, followed by trimethoprim-sulfamethoxazole 160/800mg, which guarantees good antibiotic levels in the central nervous system. Another alternative scheme, already in use and defended by some experts, is hydroxychloroquine (600mg/day) and doxycycline (200mg/day) for 12 months, with some experts recommending the lifetime use of doxycycline 200mg/day for preventing relapses [1,4,5]. The better method and interval to evaluate response to treatment and cure is not well established, but duodenal biopsy in six-month intervals has been recommended. Evaluation through non-invasive samples and PCR detection have been postulated and are being tested [1,2,15]. 

## Conclusions

Although WD was first described over a hundred years, a considerable delay in diagnosis still exists. This case report is meant to improve this entity's awareness, which is fundamental to early diagnosis and adequate treatment. The authors recommend that WD should be considered in the differential diagnosis of rheumatologic disorders, chronic abdominal pain or diarrhea, neurological manifestations not suggestive of any other specific disease, non-caseating granulomatous diseases, and cases of lymphadenopathies. The correct diagnosis could avoid unnecessary exams and treatments such as immunosuppression and improve the disease's mortality and sequels. More studies and recommendations are needed concerning screening patients and treatment, with an urgent need to improve the delay in diagnosis.
